# Infectious Diseases in Childcare Settings^1^

**DOI:** 10.3201/eid1011.040623_04

**Published:** 2004-11

**Authors:** Ralph Cordell, Larry Pickering, Frederick W. Henderson, Jody Murph

**Affiliations:** * Centers for Disease Control and Prevention, Atlanta, Georgia, USA;; † University of North Carolina at Chapel Hill, Chapel Hill, North Carolina, USA;; ‡ The Children’s Hospital of Iowa, Iowa City, Iowa, USA

**Keywords:** child daycare centers, occupational diseases, diarrhea, respiratory tract infections

In 2001, 80% of children <6 years of age were in out-of-home child care an average of 40 hours per week. More than 1.2 million persons (primarily women) were paid to care for these children in childcare centers or homes. Childcare workers are among the lowest paid segment of the workforce. They are exposed to a number of occupational hazards, including stress, injuries, and occupational exposures to infectious diseases.

## Enteric Infections

Childcare facilities are often foci for outbreaks of hepatitis A and may be linked to 14% of hepatitis A infections in the United States. Infected children are generally asymptomatic, and providers may be exposed during diaper changing. Although routine vaccination of childcare providers with hepatitis A vaccine is not currently recommended, the vaccine may be useful in protecting staff and children in the event of an outbreak.

Providers are also exposed to a variety of diarrheal illnesses. The most common causes of childcare-associated outbreaks include *Giardia*, *Cryptosporidium*, rotavirus, and other enteric viruses. Outbreaks of *Shigella* and *Escherichia coli* O147:H7 infection also occur with some frequency and are a cause for concern. Providers may also introduce infections into their own households; one study reported a 22% secondary attack rate of diarrheal illness among household contacts of childcare providers. Effective prevention and control strategies include hand hygiene, educating providers about transmission mechanisms, cleaning and disinfecting the childcare environment, and promoting vaccination among children and providers.

## Infections Posing a Risk to Fetuses of Infected Women

Cytomegalovirus is frequently transmitted in childcare settings. When infected childcare providers are pregnant, their fetuses are at risk of infection and of subsequent adverse consequences. Primary infections in pregnant women result in fetal infection 40%–50% of the time; 10% of infected infants will be symptomatic at birth, and neurologic sequelae will develop in nearly all of them. Horizontal transmission in childcare facilities may be child-to-child and child-to-adult. Simple interventions have been shown to reduce seroconversion rates in households; these interventions include avoiding kissing children on or around the mouth; avoiding sharing food, utensils, or glasses; and handwashing after diaper changes. Primary infection during pregnancy with parvovirus B19, the causative agent of erythema infectiosum, can result in hydrops fetalis or stillbirth. Prevention and control measures consist primarily of infection control, good hand hygiene, and proper disposal of tissues.

## Respiratory Infections

Although little data exist on respiratory illness in providers, correlative data give an idea of existing patterns. Although excess death due to influenza is generally associated with the elderly, on average 130–260 excess deaths occur in children <5 years of age; 66% of those deaths are in children with chronic disease. Children, however, account for most of the 21 million respiratory infections, for which medical treatment was sought, estimated to occur during an average influenza year. Schools and child care facilities play a role in sustaining outbreaks of influenza.

The viruses causing respiratory disease in childcare centers are the same ones causing disease in the community and include the following viruses: respiratory syncytial virus, parainfluenza 1-2, parainfluenza 3, influenza A, and influenza B. Influenza can be controlled with vaccination. Vaccination is recommended for a number of groups including child care providers.

